# Text phrase‐mining in identifying and classifying maternal proteins and genes across preeclampsia and similar pathologies

**DOI:** 10.14814/phy2.70262

**Published:** 2025-03-18

**Authors:** Jacqueline G. Urdang, Stephanie Masters, Nneoma Edokobi, Chitra Mukherjee, Arnib Quazi, David A. Liem, Monica Ahrens, Xuan Wang, Megan Whitham

**Affiliations:** ^1^ Virginia Tech Carilion School of Medicine Roanoke Virginia USA; ^2^ Carilion Clinic Roanoke Virginia USA; ^3^ UC Davis Davis California USA; ^4^ Virginia Tech Blacksburg Virginia USA

**Keywords:** CaseOLAP, natural language processing, preeclampsia, text phrase‐mining

## Abstract

This study aims to demonstrate that text phrase‐mining and natural language processing (NLP) can annotate huge quantities of obstetrics textual data for the discovery and evaluation of maternal protein/gene (MPG)‐disease interactions involved in the preeclampsia pathway. We employ a phrase‐mining/NLP pipeline to evaluate unique MPGs involved in six cardiovascular derangements with overlapping presentations during pregnancy. The diseases were matched with Medical Subject Headings. A textual corpus was developed from abstracts matched to these terms through PubMed. Fourty‐four MPGs were identified with respect to the diseases. Processing was performed, with unique scores for each MPG‐disease pair. Components of the score were calculated and weighted for distinctness, integrity, and popularity. Statistical analyses were conducted for the examination of protein‐disease relationships. Fourty‐four MPGs with known associations to cardiovascular disease and preeclampsia pathways were identified among the 6 diseases. MPGs shared across the greatest number of disease states were implicated in: (1) angiogenesis and vasoconstriction, (2) hemodynamic regulation, (3) hormonal regulation of metabolism, and (4) inflammation. NLP and text phrase‐mining are successfully applied to Obstetrics abstracts with accuracy and speed. This approach holds promise in synthesizing large volumes of data for presenting trends in the Obstetric literature and for the identification of promising biomarkers.

## Graphical abstract

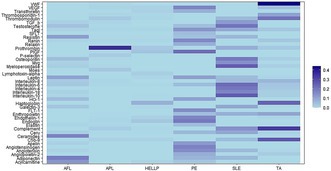

## 1 INTRODUCTION

The current enthusiasm for the development of artificial intelligence (AI) techniques to address unanswered challenges in Obstetrics (OB) and Gynecology has been recently stressed (Shazly et al., 2022). One area in which AI has been suggested as a possible tool is in addressing the recent rapid growth and expansion of publications that outpace the rate at which manual review can occur. Text phrase‐mining and natural language processing approaches may allow evaluation of disease relationships within this vast quantity of unstructured text data in a reduced amount of time (Voskamp et al., 2022). Specifically, this tool may reconfigure unstructured text to a format from which meaningful patterns can be drawn.

Text mining of existing peer‐reviewed publications has been used in multiple applications in the medical field; including classification of disease phenotypes (Delude, 2015; Liem et al., 2018; Vuori et al., 2023), description of drug‐gene interactions (Anand et al., 2022), identification of cancer biomarkers (Li et al., 2023), and synthesis of behavioral factors associated with disease (Brscic et al., 2021). Recently, Liem et al. performed a comprehensive examination of PubMed abstracts to classify distinct extracellular matrix protein (ECM) profiles implicated in the development of 6 cardiovascular disease profiles. They used a novel phrase‐mining pipeline called Context‐Aware Semantic Online Analytical Processing (CaseOLAP) to score ECM proteins according to their “integrity,” “popularity,” and “distinctiveness,” and were able to demonstrate that a combination of phrase‐mining algorithms and a “large‐scale network can effectively annotate large volumes of cardiovascular disease textual data for relationship discovery and extraction of meaningful insights” (Liem et al., 2018).

Hypertensive and cardiovascular disorders of pregnancy are some of the most commonly occurring complications of pregnancy (Wu et al., 2023). The Centers for Disease Control and Prevention reports that the prevalence of pregnancy‐associated hypertension has increased from 10.8% in 2017 to a rate of 13.0% in 2019, while the prevalence of chronic hypertension has increased from 2.0% to 2.3% (Ford et al., 2022). Among maternal deaths that occurred during delivery hospitalization, 31.6% had any hypertensive disorder in pregnancy documented, and 24.3% of which were pregnancy‐associated (Ford et al., 2022).

Hypertensive disorders of pregnancy can have unique defining features (such as in HELLP) or can share overlapping clinical phenotypes with different cardiovascular derangements when presenting during the pregnancy time period (such as in antiphospholipid antibody syndrome (APL), systemic lupus erythematosus (SLE), or with thrombotic angiopathies (TA)). These disease types can have similar presentations but distinct treatments and cardiovascular implications. Given the association between hypertensive disorders of pregnancy and cardiovascular pathophysiologies, we decided to evaluate cardiovascular maternal proteins and genes (MPG) involved in subtypes of cardiovascular derangements that can have overlapping clinical presentations with preeclampsia. These were chosen as the disease states of interest (DOI) by an expert in the field: preeclampsia, HELLP syndrome, acute fatty liver of pregnancy, thrombotic angiopathies, antiphospholipid syndrome, and SLE. This set of cardiovascular and pregnancy‐specific disease types was selected as an ideal starting point for textual analysis in the Obstetric field due to their manageable size—of fewer than 10 but greater than a negligible number below 3—and their known overlap with maternal phenotypic presentation, making them a suitable use case for the application of text‐mining tools.

The previously mentioned text phrase‐mining/natural language processing pipeline with CaseOLAP was used to assess associations between the MPGs and the six DOIs. These were semantically scored according to their “integrity,” “popularity,” and “distinctiveness” with regard to the six DOIs using the CaseOLAP pipeline. Our analyses not only validated existing relationships between MPG proteins and diseases but also highlighted novel protein–protein and protein–disease associations, as well as unique protein identifiers. Our study provides support for adopting previously underutilized literature mining methods in Obstetric research to synthesize associations of risk factors with disease states of interest for future experimental or clinical study.

## 2 MATERIALS AND METHODS

### 2.1 Selection of diseases of interest

Six diseases with hypertensive and cardiovascular diseases of pregnancy were chosen as the diseases of interest (DOI) and focus of this study. These complications include (PE), HELLP syndrome (hemolysis, elevated liver enzymes, and low platelet count), AFL, TA, APL, and SLE. The selection was based on their clinical significance, with overlapping phenotypes, and relevance to maternal health (Hammoud & Ibdah, 2014; Rhein et al., 2024; Urra et al., 2024).

### 2.2 Text phrase‐mining data collection

To comprehensively review the existing literature on the selected DOIs, we employed the Medical Subject Headings (MeSH) system for the development of our textual corpus. MeSH is a controlled vocabulary thesaurus developed and maintained by the National Library of Medicine (NLM) in the United States (National Institutes of Health, 2024). MeSH terms are organized in a hierarchical tree structure, providing a systematic and organized representation of medical concepts. Each DOI was matched with the MeSH hierarchy, ensuring a standardized approach to categorizing and organizing information. The MeSH descriptors for the selected pregnancy‐related complications and comorbidities are detailed in (Table 1). Each descriptor is associated with a specific MeSH code within the hierarchy. Using the identified MeSH descriptors and codes, a systematic literature search was conducted across the PubMed databases. A total of 184,235 abstracts were retrieved from PubMed for all six DOIs. Out of the total 184,235 unique abstracts, we were able to retrieve 34,925 on PE, 1960 on HELLP, 43,410 on AFL, 24,709 on TA, 9092 on APL, and 69,280 on SLE.

**TABLE 1:** Medical subject heading (MeSH) descriptors for pregnancy‐related complications.

MeSH descriptors	Tree number
Pre‐eclampsia	C12.050.703.395.249
HELLP syndrome	C12.050.703.395.186
Hypertension, pregnancy‐induced	C12.050.703.395 C14.907.489.480
Eclampsia	C12.050.703.395.124
Reye syndrome	C06.552.241.649 C10.228.140.163.780 C18.452.132.780
Fatty liver	C06.552.241
Non‐alcoholic fatty liver disease	C06.552.241.519
Fatty liver, alcoholic	C06.552.241.390 C06.552.645.390 C25.775.100.087.645.390
Hemolytic‐uremic syndrome	C12.050.351.968.419.936.463 C12.200.777.419.936.463 C12.950.419.936.463 C15.378.050.141.610 C15.378.140.855.925.500 C15.378.243.937.925.500
Purpura, thrombocytopenic, idiopathic	C15.378.100.802.687.600 C15.378.140.855.925.750.600 C15.378.243.937.925.750.600 C15.378.463.740 C20.111.759 C20.841.600 C23.550.414.950.687.600 C23.888.885.687.687.600
Thrombotic microangiopathies	C15.378.140.855.925 C15.378.243.937.925
Purpura, thrombocytopenic	C15.378.100.802.687 C15.378.140.855.925.750 C15.378.243.937.925.750 C20.841 C23.550.414.950.687 C23.888.885.687.687
Atypical hemolytic uremic syndrome	C12.050.351.968.419.936.463.500 C12.200.777.419.936.463.500 C12.950.419.936.463.500 C15.378.050.141.610.500 C15.378.140.855.925.500.500 C15.378.243.937.925.500.500
Purpura, thrombotic thrombocytopenic	C15.378.100.802.687.680 C15.378.140.855.925.750.680 C15.378.243.937.925.750.680 C15.378.925.850 C23.550.414.950.687.680 C23.888.885.687.687.680
Antiphospholipid syndrome	C20.111.197
Lupus vasculitis, central nervous system	C01.207.245.550.500 C01.207.570.450 C10.114.875.850 C10.228.140.300.850.750 C10.228.140.430.550.500 C10.228.228.245.550.500 C10.228.228.570.450 C10.586.250.550.500 C10.586.625.500.500 C14.907.253.946.850 C14.907.940.907.850 C17.300.480.750 C20.111.258.962.900 C20.111.590.750
Lupus erythematosus, cutaneous	C17.300.475 C17.800.480
Lupus erythematosus, systemic	C17.300.480 C20.111.590
Panniculitis, lupus erythematosus	C17.300.475.479.400 C17.300.710.400 C17.800.480.479.400 C17.800.566.400
Lupus erythematosus, discoid	C17.300.475.479 C17.800.480.479
Lupus nephritis	C12.050.351.968.419.570.363.680 C12.200.777.419.570.363.680 C12.950.419.570.363.680 C17.300.480.680 C20.111.590.560

254 MPGs were selected for text phrase‐mining analysis (Appendix S1). MPGs were chosen by current practicing physician experts in the field of Obstetrics and Gynecology; some are known to be highly linked to CV diseases, with some being previously associated with PE. Of the 254 selected MPGs, 44 were detected to have been mentioned within the abstracts curated for analysis, from which natural language processing with CaseOLAP analysis was performed.

### 2.3 Algorithm application

**FIGURE 1:**
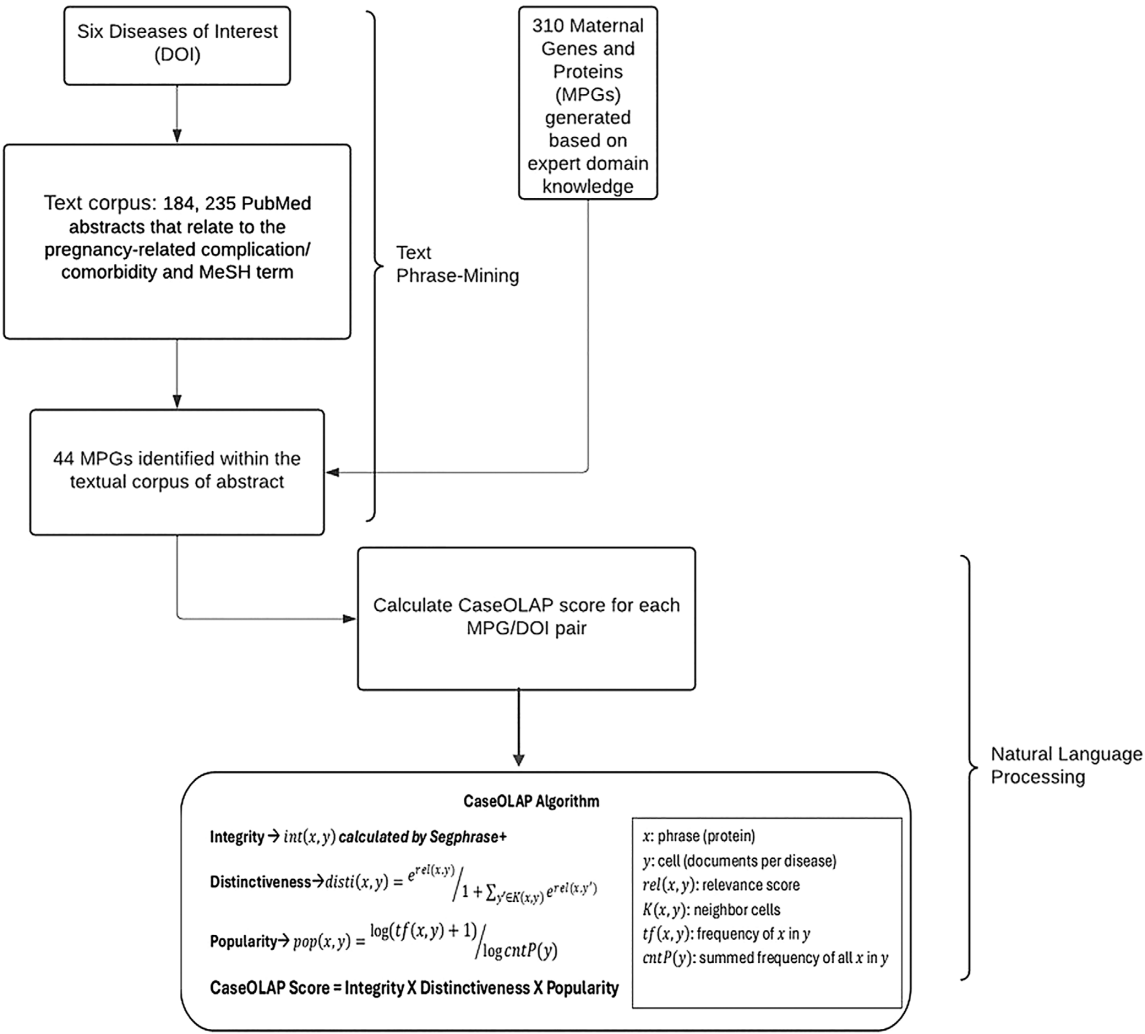
Workflow and Algorithm. Demonstrates the application of the text phrase‐mining, natural language processing pipeline to the Obstetric literature for the Diseases of Interest (DOIs) and Maternal Proteins and Genes (MPGs). Illustrating how the six diseases of interest were processed with respect to the maternal proteins and genes of interest, ultimately providing us with disease‐protein or disease‐gene pairings on which to use natural language processing. Arrows indicate the chronology of data processing.

We conducted a phrase‐mining analysis utilizing the CaseOLAP (Liem et al., 2018; Tao et al., 2016) algorithm to assess the associations between the 44 MPGs associated with collected abstracts and each of the six DOIs. This analysis was carried out within the framework of the constructed workflow (Figure 1). The text corpus, comprising 184,235 abstracts, was partitioned into six groups corresponding to the six DOIs. Following this step, the CaseOLAP algorithm was employed to quantify associations between MPGs and DOIs, producing a score ranging from 0.0 to 1.0 for each MPG‐DOI pairing. A higher CaseOLAP score indicates a stronger association between the MPG and the corresponding DOI. Specifically, the CaseOLAP algorithm integrates three key components in its assessments: integrity, distinctiveness, and popularity. The final CaseOLAP score for each pair of MPG and DOI is the product of the three components. The CaseOLAP scores enable quantitative analysis and interpretation of otherwise highly complex linkages between MPG and DOI via mining of vast amounts of textual data from PubMed.

The integrity score measures the coherent and meaningful semantic units inside the textual corpora. Finding essential semantic pieces that, together, represent meaningful concepts is the goal of this component. The generated list of MPGs was curated by an expert in Maternal Fetal Medicine, such that integrity scoring was set to be the same across all MPGs (0.8).

The term “distinctiveness” highlights the specificity and originality of terms or entities in the dataset. This tenet seeks to identify unique components that support the information structure as a whole. For example, if MPG X is referenced in multiple abstracts for multiple disease states, MPG X will receive a score closer to 0, representing low distinctiveness. However, if MPG X is referenced in multiple abstracts for only one disease state, then MPG X will receive a score closer to 1, representing high distinctiveness.

Popularity considers how frequently or widely a term or entity appears in the dataset. Popularity considers a particular element's prevalence as a sign of its importance within the data. If distinctiveness represents how frequently MPG X is mentioned in multiple abstracts across multiple disease states, then popularity seeks to determine how often MPG X is mentioned within one specific DOI, with values closer to 1 indicating higher popularity for that MPG‐DOI pairing and vice versa.

### 2.4 Data analysis

Statistical analyses were performed using the R programming language on R version v4.4.2 (link to code repository available below). We performed descriptive and visual data analyses to study the relationships between the 44 MPGs and the 6 DOIs, including heat map, count plot, and bar plots. A principal components (PC) analysis (PCA) was performed on the scores to examine the similarities in the MPGs shared across DOIs.

## 3 RESULTS

**FIGURE 2:**
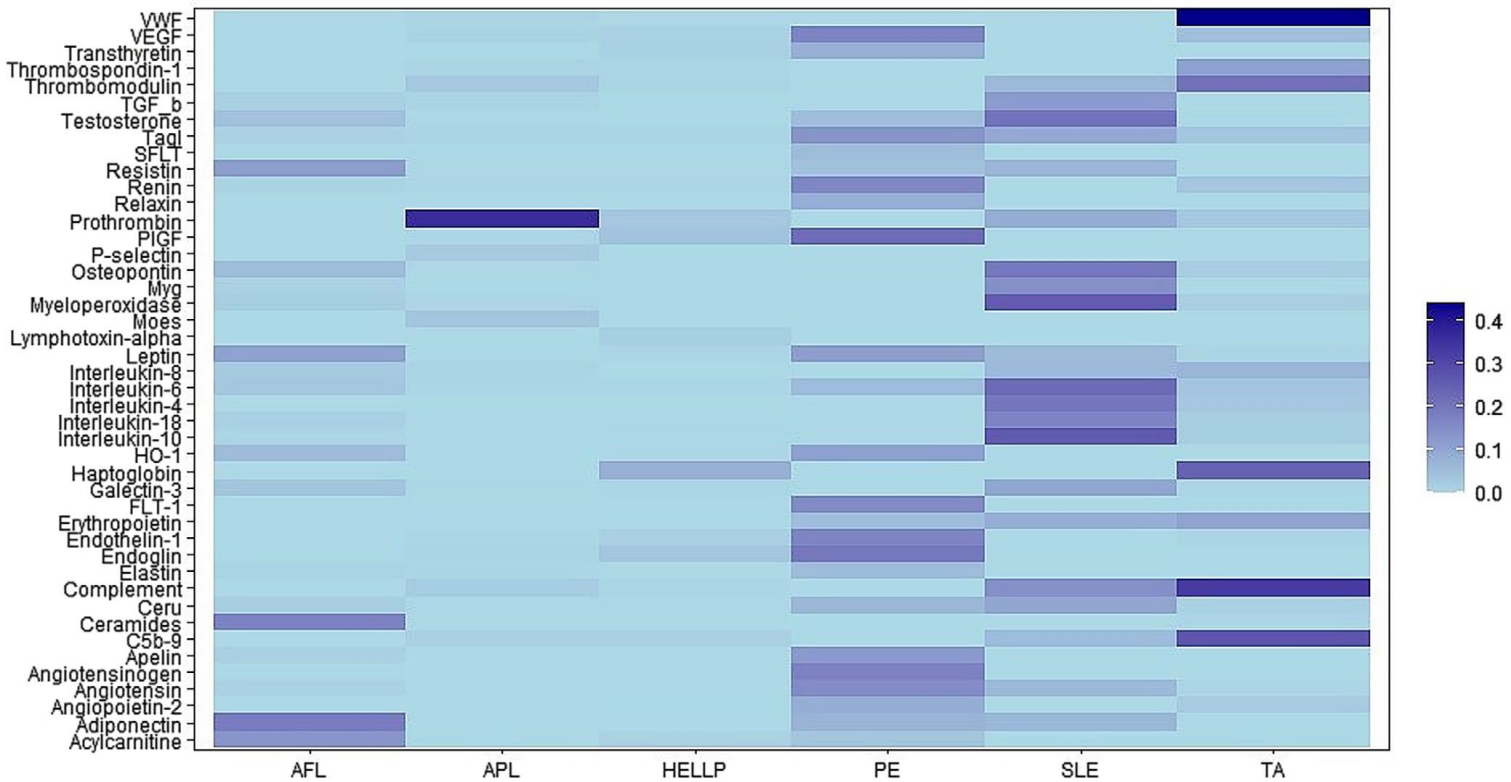
Heat Map of Disease‐Protein and Disease‐Gene Associations. Context‐aware Semantic Online Analytical Processing (CaseOLAP) scores are represented across the Maternal Proteins and Genes (MPGs) on the *x*‐axis and the six Diseases of Interest (DOIs) on the *y*‐axis. Higher scores are depicted by darker cells.

By applying text phrase‐mining to publicly available PubMed abstracts, we extracted 184,235 abstracts containing MeSH descriptors of our 6 DOIs: PE, TA, HELLP, AFL, APL, and SLE. By applying text mining to our textual corpus and 254 MPGs of interest, we were able to identify 44 MPGs with relationships to the DOIs (Table 2). These MPGs were scored using CaseOLAP. Table 3 presents the MPG alongside its corresponding CaseOLAP score generated by the algorithm, including individual scores for the integrity, distinctiveness, and popularity components. Results of this scoring are represented as a heat map (Figure 2) and their scoring patterns are highlighted, where higher CaseOLAP scores are represented by darker cells. The distribution is heterogeneous, demonstrating varied profiles of MPG–sease relationships across the six disease states.

**TABLE 2:** Maternal proteins and genes.

Maternal protein/genes		
Acylcarnitine	Galectin‐3	p‐selectin
Adiponectin	Haptoglobin	Placental growth factor (PIGF)
Angiopoietin‐2	Heme oxygenase‐1 (HO‐1)	Prothrombin
Angiotensin	Interleukin‐10	Relaxin
Angiotensinogen	Interleukin‐18	Renin
Apelin	Interleukin‐4	Resistin
C5b‐9 (membrane attack complex)	Interleukin‐6	Soluble FMS‐like tyrosine kinase (SFLT)
Ceramides	Interleukin‐8	Transgelin (tag)
Ceruloplasmin	Leptin	Testosterone
Complement	Lymphotoxin‐alpha	Transforming growth factor_b (TFG_b)
Elastin	Moesin	Thrombomodulin
Endoglin	Myeloperoxidase	Thrombospondin‐1
Endothelin‐1	Myoglobin	Transthyretin
Erythropoietin	Vascular endothelial growth factor (VEGF)	Vascular endothelial growth factor receptor‐1 (FLT‐1)
Osteopontin	von Willebrand factor (vWF)	

**TABLE 3:** Context‐aware semantic online analytical processing (CaseOLAP) score for each maternal protein or gene by disease.

Phrase	Score	Popularity	Integrity	Distinct
CaseOLAP preeclampsia				
Acylcarnitine	0.04	0.24	0.8	0.18
Adiponectin	0.07	0.44	0.8	0.20
Angiopoietin‐2	0.08	0.29	0.8	0.36
Angiotensin	0.14	0.49	0.8	0.35
Angiotensinogen	0.17	0.47	0.8	0.46
Apelin	0.12	0.36	0.8	0.42
Ceru	0.07	0.36	0.8	0.24
Elastin	0.06	0.25	0.8	0.29
Endoglin	0.21	0.60	0.8	0.43
Endothelin‐1	0.17	0.54	0.8	0.40
Erythropoietin	0.05	0.36	0.8	0.18
FLT‐1	0.17	0.41	0.8	0.51
HO‐1	0.11	0.42	0.8	0.33
Interleukin‐10	0.03	0.37	0.8	0.12
Interleukin‐6	0.05	0.47	0.8	0.15
Interleukin‐8	0.04	0.28	0.8	0.17
Leptin	0.12	0.53	0.8	0.28
PIGF	0.16	0.43	0.8	0.46
PlGF	0.23	0.67	0.8	0.44
Relaxin	0.09	0.26	0.8	0.41
Renin	0.16	0.59	0.8	0.35
Resistin	0.04	0.31	0.8	0.18
SFLT	0.06	0.16	0.8	0.44
Tagl	0.14	0.59	0.8	0.29
Testosterone	0.05	0.39	0.8	0.16
Thrombomodulin	0.04	0.42	0.8	0.11
Transthyretin	0.08	0.30	0.8	0.33
VEGF	0.18	0.64	0.8	0.35
CaseOLAP HELLP syndrome				
Acylcarnitine	0.01	0.17	0.8	0.09
Angiotensin	0.00	0.11	0.8	0.01
C5b‐9	0.01	0.32	0.8	0.06
Ceru	0.00	0.11	0.8	0.02
Complement	0.01	0.37	0.8	0.02
Endoglin	0.03	0.42	0.8	0.10
Endothelin‐1	0.02	0.34	0.8	0.06
Galectin‐3	0.00	0.11	0.8	0.04
Haptoglobin	0.08	0.47	0.8	0.21
HO‐1	0.00	0.11	0.8	0.02
Interleukin‐10	0.00	0.17	0.8	0.02
Interleukin‐6	0.00	0.25	0.8	0.02
Leptin	0.00	0.25	0.8	0.02
Lymphotoxin‐alpha	0.02	0.11	0.8	0.23
Myeloperoxidase	0.00	0.11	0.8	0.01
PIGF	0.01	0.17	0.8	0.05
PlGF	0.04	0.49	0.8	0.10
Prothrombin	0.03	0.52	0.8	0.08
Renin	0.00	0.25	0.8	0.02
Resistin	0.00	0.11	0.8	0.02
Tagl	0.01	0.32	0.8	0.03
Thrombomodulin	0.01	0.27	0.8	0.03
Thrombospondin‐1	0.00	0.11	0.8	0.05
Transthyretin	0.01	0.17	0.8	0.10
VEGF	0.01	0.38	0.8	0.04
vWF	0.00	0.17	0.8	0.01
CaseOLAP acute fatty liver				
Acylcarnitine	0.14	0.38	0.8	0.45
Adiponectin	0.19	0.60	0.8	0.39
Angiotensin	0.01	0.32	0.8	0.05
Apelin	0.02	0.22	0.8	0.09
Ceramides	0.17	0.40	0.8	0.55
Ceru	0.02	0.29	0.8	0.09
Elastin	0.01	0.15	0.8	0.09
Galectin‐3	0.04	0.27	0.8	0.17
HO‐1	0.05	0.40	0.8	0.17
Interleukin‐10	0.01	0.29	0.8	0.04
Interleukin‐18	0.01	0.22	0.8	0.08
Interleukin‐6	0.03	0.47	0.8	0.08
Interleukin‐8	0.03	0.29	0.8	0.11
Leptin	0.11	0.59	0.8	0.23
Methamphetamine	0.01	0.09	0.8	0.13
Myeloperoxidase	0.02	0.34	0.8	0.07
Myg	0.01	0.15	0.8	0.10
Osteopontin	0.05	0.36	0.8	0.18
Renin	0.01	0.38	0.8	0.03
Resistin	0.12	0.44	0.8	0.34
Tagl	0.01	0.41	0.8	0.04
Testosterone	0.05	0.43	0.8	0.13
TGF_b	0.02	0.15	0.8	0.13
CaseOLAP thrombotic angiopathies				
Angiopoietin‐2	0.02	0.20	0.8	0.15
Angiotensin	0.00	0.20	0.8	0.02
C5b‐9	0.27	0.56	0.8	0.61
Ceramides	0.00	0.09	0.8	0.04
Ceru	0.01	0.22	0.8	0.07
Complement	0.33	0.72	0.8	0.57
Endothelin‐1	0.01	0.26	0.8	0.03
Erythropoietin	0.11	0.41	0.8	0.32
Galectin‐3	0.00	0.09	0.8	0.04
Haptoglobin	0.24	0.56	0.8	0.54
Interleukin‐10	0.02	0.32	0.8	0.08
Interleukin‐18	0.02	0.22	0.8	0.12
Interleukin‐4	0.03	0.28	0.8	0.14
Interleukin‐6	0.04	0.42	0.8	0.11
Interleukin‐8	0.07	0.33	0.8	0.26
Leptin	0.01	0.27	0.8	0.03
Myeloperoxidase	0.02	0.28	0.8	0.07
Osteopontin	0.02	0.24	0.8	0.10
Prothrombin	0.03	0.51	0.8	0.07
Renin	0.03	0.43	0.8	0.10
Tagl	0.04	0.45	0.8	0.10
Thrombomodulin	0.21	0.55	0.8	0.47
Thrombospondin‐1	0.11	0.32	0.8	0.44
VEGF	0.05	0.50	0.8	0.13
vWF	0.44	0.67	0.8	0.82
CaseOLAP antiphospholipid syndrome				
C5b‐9	0.01	0.34	0.8	0.05
Ceru	0.00	0.09	0.8	0.02
Complement	0.02	0.53	0.8	0.05
Elastin	0.00	0.09	0.8	0.06
Endoglin	0.01	0.30	0.8	0.03
Endothelin‐1	0.00	0.24	0.8	0.02
Erythropoietin	0.00	0.15	0.8	0.02
Galectin‐3	0.00	0.09	0.8	0.04
Interleukin‐6	0.00	0.26	0.8	0.02
Interleukin‐8	0.01	0.18	0.8	0.05
Moes	0.04	0.15	0.8	0.30
Myeloperoxidase	0.00	0.18	0.8	0.02
Osteopontin	0.00	0.09	0.8	0.02
p‐selectin	0.02	0.09	0.8	0.32
PlGF	0.00	0.30	0.8	0.01
Prothrombin	0.36	0.83	0.8	0.55
Renin	0.00	0.29	0.8	0.02
Resistin	0.00	0.09	0.8	0.02
Tagl	0.01	0.37	0.8	0.03
Testosterone	0.00	0.15	0.8	0.01
TGF_b	0.01	0.09	0.8	0.09
Thrombomodulin	0.03	0.43	0.8	0.09
Thrombospondin‐1	0.01	0.15	0.8	0.06
VEGF	0.01	0.40	0.8	0.03
vWF	0.01	0.33	0.8	0.02
CaseOLAP systemic lupus erythematosus				
Adiponectin	0.06	0.42	0.8	0.19
Angiotensin	0.06	0.40	0.8	0.18
C5b‐9	0.06	0.42	0.8	0.16
Ceru	0.10	0.38	0.8	0.33
Complement	0.15	0.65	0.8	0.28
Erythropoietin	0.08	0.38	0.8	0.27
Galectin‐3	0.10	0.31	0.8	0.40
Interleukin‐10	0.26	0.53	0.8	0.62
Interleukin‐18	0.17	0.38	0.8	0.55
Interleukin‐4	0.20	0.43	0.8	0.58
Interleukin‐6	0.22	0.58	0.8	0.48
Interleukin‐8	0.06	0.31	0.8	0.24
Leptin	0.06	0.45	0.8	0.16
Myeloperoxidase	0.26	0.51	0.8	0.63
Myg	0.15	0.30	0.8	0.62
Osteopontin	0.19	0.42	0.8	0.58
Prothrombin	0.08	0.60	0.8	0.18
Resistin	0.07	0.33	0.8	0.25
Schizophrenia	0.15	0.40	0.8	0.48
Tagl	0.09	0.53	0.8	0.22
Testosterone	0.21	0.50	0.8	0.52
TGF_b	0.12	0.26	0.8	0.58
Thrombomodulin	0.06	0.44	0.8	0.17

**FIGURE 3:**
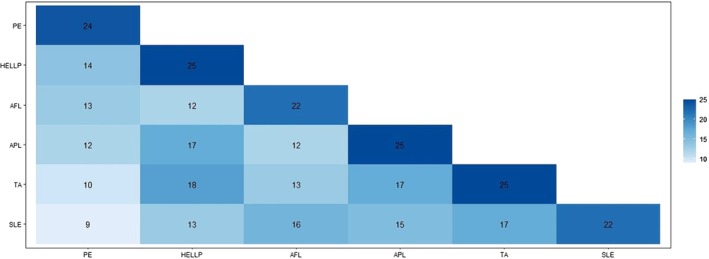
Count Plot Heat Map of MPGs Common to Two Diseases. Maternal Proteins and Genes (MPGs) found in each disease or labeled as PE (preeclampsia) (*n* = 24), HELLP (HELLP syndrome) (*n* = 25), AFL (acute fatty liver) (*n* = 22), APL (antiphospholipid syndrome) (*n* = 25), TA (thrombotic angiopathies) (*n* = 25), and SLE (systemic lupus erythematosus) (*n* = 22).

**FIGURE 4:**
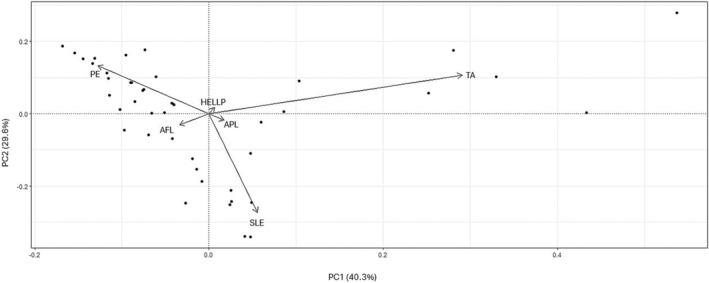
Principal Component Analysis on MPGs over all 6 DOIs. The scatterplot was produced by taking the two principal components (PC) of maximum variance. PC1 and PC2 accounted for 69.9% of the variance. Each dot on the PC plane represents an individual MPG, and the arrowhead vectors are the projections of the six DOIs. Based on the two PCs displayed, we observe that the vectors are separated, indicating disparate MPG scores between the DOIs.

**FIGURE 5:**
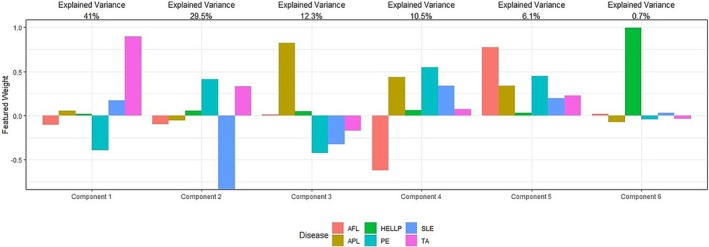
Bar Graph of DOIs to the Principal Component. This figure shows the representative contribution of each DOI to each of the six principal components. Each vertical bar represents the weight of the specific DOI to the indicated PC. The first two components (PC1 and PC2) were used to construct the scatterplot in Figure 4.

To compare the overlap and similarities of the identified MPG proteins between DOIs, we developed a boxplot matrix of the counts of proteins in common with the disease states of interest (Figure 3). There were 28 proteins associated with PE, 26 associated with HELLP, 22 associated with AFL, 25 associated each with APL and TA, and 25 proteins associated with TA. Further, MPG proteins common to two diseases are shown. The largest overlap in shared MPG proteins between two DOIs was seen between TA and HELLP (*n* = 18). A principal component analysis (PCA) was performed, analyzing all CaseOLAP scores for the 6 DOIs. The scatterplot (Figure 4) displays the first two principal components (PC). Each PC is a linear combination of all six DOIs. The representative contribution of each individual disease state of interest or feature to each of the six PCs is shown (Figure 5). Each vertical bar in Figure 5 represents the feature weight of a specific disease state to the indicated PC. PC1 and PC2 accounted for 69.4% of the total variance. Each dot on the PC plane represents an individual protein, and the arrowhead vectors are the projections of the PCs for each of the six disease states. Based on the two PCs displayed, we observed that all the vectors are separated, indicating divergence in the overall protein scoring patterns for all six DOIs. These results point to a lack of shared biological functions of the MPG in the pathophysiology of hypertensive and cardiovascular disorders.

Several proteins displayed strong associations with TA, SLE, PE, and AFL. Of particular note and interest, the protein with the strongest relationship to PE was placental growth factor (PIGF).

The vWF protein displayed the strongest association with any of the DOIs, specifically TA. In addition to vWF, the proteins most strongly associated with TA were Thrombomodulin and Haptoglobin—mediators in hemostasis—as well as Complement, particularly end complement (C5b‐9): responsible for the immune response. Testosterone, Osteopontin, Myeloperoxidase, and Complement exhibited the strongest associations with SLE. The protein displaying the strongest associations to AFL was Acylcarnitine. The other strongest association to AFL was Ceramides. Although these are sphingolipids rather than proteins, they are included here as products of gene expression.

## 4 STRUCTURED DISCUSSION

### 4.1 Principal findings

Maternal proteins and genes (MPG) are known to play important roles in the genesis and progression of a wide spectrum of hypertensive and cardiovascular diseases of pregnancy (Williams & Broughton Pipkin, 2011). Further, studies have begun to recognize certain MPG as potential clinical biomarkers (Schuermans et al., 2024). This study found clear relationships between MPG and six disease states. Certain DOIs, for example, APL, HELLP, and TA, share groups of associated proteins (Figure 3), indicating a common mechanistic pathogenesis underlying these disease phenotypes, consistent with prior research. In contrast, our novel findings TA and PE demonstrate distinct groups of protein associations, implying unique molecular pathways (Figure 4). In this way, we were able to confirm previously held knowledge about certain MPG‐MPG relationships to PE and diseases with overlapping clinical phenotypes. For instance, HELLP and TA seem to share many MPGs in common, indicating a common pathogenesis pathway.

### 4.2 Results in the context of what is known

We identify several potential protein targets that stand out as outliers distinct to different disease processes with overlapping phenotypes. Our analysis provides support that PIGF and endoglin are distinctly associated with the PE disease pathway. A recent report showed that women hospitalized with a hypertensive disorder of pregnancy were at greater risk of developing PE with severe features within 2 weeks if they had ratios of fms‐like tyrosine kinase 1 (sFLT‐1) to placental growth factor (PIGF) (serum sFlt‐1:PIGF ratio) >40 (Thadhani et al., 2022). Further, a transcriptomic survey of cells found sFLT1/placental growth factor (PGF) to be dysregulated in transcription in the syncytium in early PE (Inbal et al., 2023). This finding lends support to our methodology of text phrase‐mining as a potential tool for identifying biomarkers in OB. Our analysis also highlights that TA (Gavriilaki et al., 2019) are uniquely associated with vWF and complement (particularly C5b‐9). These associations are supported by the literature, and the text phrase‐mining approach highlights that vWF and complement are truly uniquely associated with TAs such that levels of these proteins might be considered for evaluation to support their diagnoses where there is phenotypic overlap. Alternatively, SLE was found to be most strongly associated with MPO and IL‐10. MPO, a marker of leukocyte recruitment, is associated with the pathogenesis of a vascular disorders and inflammatory conditions like SLE (Heidari et al., 2016). IL‐10, a regulator of the inflammatory response, has been demonstrated as being deeply dysregulated in SLE patients (Rzeszotarska et al., 2022). Immunohistochemical analysis has also confirmed elevated levels of MPO in placental sections of PE patients (Gandley et al., 2008). Previous studies have noted a significant difference in the MPO level of plasma and placental extracts between PE samples and healthy women (Hung et al., 2012). MPO might be considered for further study in patients with SLE to evaluate this population's risk of developing PE. Similarly, the proteins adiponectin and resistin were outliers on the AFL vector. This follows as serum levels of adiponectin have been found to be decreased in obesity, and a negative association between serum levels of adiponectin and liver enzyme levels has been shown in healthy subjects (Finelli & Tarantino, 2013). Adiponectin and resistin may thus be targets of evaluation as biomarkers specific to this disease process in cases presenting clinical diagnostic conundrums for this condition to distinguish the condition from HELLP.

### 4.3 Clinical implications

Text phrase‐mining and natural language processing with the use of the CaseOLAP algorithm can be used for similar evaluations of the OB literature that may require the processing of large quantities of data for the discovery of relationships. Using these processes, critical relationships between health factors and disease states can be described succinctly. Future directions of our research team include the use of natural language processing approaches on patient‐level physician notes exploring relationships between health features and disease presentations at a more granular level of detail.

The ability of data mining to expand upon known and to identify new relationships is ever‐changing and growing. Further research in this field could drive investigation and review of other disease states of interest to the OB field (e.g., premature rupture of membranes (PROM and PPROM), pre‐term labor (PTL), and maternal mortality).

### 4.4 Research implications

The CaseOLAP algorithm approach used in this study serves as a model of text phrase‐mining to reduce time spent on manual review of the literature in synthesizing and highlighting critical relationships. The approach also eliminates human error in such literature reviews that may come from missing essential abstracts, protein or gene references, or prior knowledge of disease processes. This study is a preliminary example of text mining application to evaluate disease relationships within the OB field.

### 4.5 Strengths and limitations

Despite the previously mentioned benefits of the CaseOLAP algorithm, there are limitations and methodological considerations that must be considered. The CaseOLAP system cannot distinguish between differences in data sets (e.g., text, imaging, or numerical data). To avoid confusion and error with mixed data presentation, our study made use exclusively of first target abstracts with almost universal text data. This approach may result in the loss of important data, but this approach reduces semantic complexity and minimizes inaccurate protein‐disease relationships. Another limitation is identified with the inclusion of review article abstracts in our textual corpora, which may falsely increase the popularity score as calculated using CaseOLAP; however, we felt the inclusion of these studies was important in identifying the breadth of MPGs that may be noted in the existing abstract literature. Further, the CaseOLAP score cannot demonstrate directionality of whether the protein is up‐ or downregulated in the DOI. This is a limitation that must be mentioned when discussing the role of proteins in the pathogenesis of diseases. Further, this limitation could potentially impact calculations within data analysis. For example, if sources vary between findings of up‐ or downregulation, a calculation may not account for direction and will result in a strong relationship when the relationship might, in fact, be weak. It is unlikely that such a case occurred in this study, but the possibility exists and must be recognized here. Another limitation lies in the popularity score calculated by the CaseOLAP algorithm. A lack of prior research and known associations between a protein and a disease will inherently result in fewer follow‐up studies with fewer publications. Therefore, a popularity score may be lower, introducing bias. A popularity score closer to 0 does not necessitate a lack of relationship.

## 5 CONCLUSIONS

Our text phrase‐mining, natural language processing pipeline was able to mine great amounts of data with accuracy, speed, and consistency in evaluating DOIs involved in common OB complications and presentations. We believe this approach holds great promise in synthesizing large volumes of data for presenting trends and updates to the OB literature.

## FUNDING INFORMATION

This work was supported by a 2023 grant from the Fralin Biomedical Research Institute Center for Health Behaviors Research.

## CONFLICT OF INTEREST STATEMENT

The authors report no conflict of interest.

## ETHICS APPROVAL STATEMENT

The study was IRB approved under Carilion Clinic IRB #22–1810, and a formal waiver of consent was granted.

## Supporting information


Appendix S1.

## Data Availability

The data that support the findings of this study are available from the corresponding author upon reasonable request.

## References

[phy270262-bib-0001] Anand, S. , Iyyappan, O. R. , Manoharan, S. , Anand, D. , Jose, M. A. , & Shanker, R. R. (2022). Text mining protocol to retrieve significant drug‐gene interactions from PubMed abstracts. Methods in Molecular Biology, 2496, 17–39. 35713857 10.1007/978-1-0716-2305-3_2

[phy270262-bib-0002] Brscic, M. , Contiero, B. , Schianchi, A. , & Marogna, C. (2021). Challenging suicide, burnout, and depression among veterinary practitioners and students: Text mining and topics modelling analysis of the scientific literature. BMC Veterinary Research, 17, 294. 34488757 10.1186/s12917-021-03000-x PMC8419380

[phy270262-bib-0003] Delude, C. M. (2015). Deep phenotyping: The details of disease. Nature, 527, S14–S15. 26536218 10.1038/527S14a

[phy270262-bib-0004] Finelli, C. , & Tarantino, G. (2013). What is the role of adiponectin in obesity related non‐alcoholic fatty liver disease? World Journal of Gastroenterology, 19(6), 802–812. 23430039 10.3748/wjg.v19.i6.802 PMC3574877

[phy270262-bib-0005] Ford, N. D. , Cox, S. , Ko, J. Y. , Ouyang, L. , Romero, L. , Colarusso, T. , Ferre, C. D. , Kroelinger, C. D. , Hayes, D. K. , & Barfield, W. D. (2022). Hypertensive disorders in pregnancy and mortality at delivery hospitalization – United States, 2017–2019. Morbidity and Mortality Weekly Report, 71, 585–591. 35482575 10.15585/mmwr.mm7117a1 PMC9098235

[phy270262-bib-0006] Gandley, R. E. , Rohland, J. , Zhou, Y. , Shibata, E. , Harger, G. F. , Rajakumar, A. , Kagan, V. E. , Markovic, N. , & Hubel, C. A. (2008). Increased myeloperoxidase in the placenta and circulation of women with preeclampsia. Hypertension, 52, 387–393. 18591459 10.1161/HYPERTENSIONAHA.107.107532 PMC2735015

[phy270262-bib-0007] Gavriilaki, E. , Anagnostopoulos, A. , & Mastellos, D. C. (2019). Complement in thrombotic Microangiopathies: Unraveling Ariadne's thread into the labyrinth of complement therapeutics. Frontiers in Immunology, 10, 337. 30891033 10.3389/fimmu.2019.00337 PMC6413705

[phy270262-bib-0008] Hammoud, G. M. , & Ibdah, J. A. (2014). Preeclampsia‐induced liver dysfunction, HELLP syndrome, and acute fatty liver of pregnancy. Clinical Liver Disease, 4(3), 69–73. 10.1002/cld.409 30992924 PMC6448736

[phy270262-bib-0009] Heidari, Z. , Mahmoudzadeh Sagheb, H. , & Sheibak, N. (2016). Immunohistochemical expression of myeloperoxidase in placental samples of systematic lupus erythematosus pregnancies. Journal of Family & Reproductive Health, 10(2), 64–70. 27648095 PMC5026670

[phy270262-bib-0010] Hung, T.‐H. , Chen, S.‐F. , Lo, L.‐M. , Li, M.‐J. , Yeh, Y.‐L. , & Hsieh, T.‐T. (2012). Myeloperoxidase in the plasma and placenta of normal pregnant women and women with pregnancies complicated by preeclampsia and intrauterine growth restriction. Placenta, 33, 294–303. 22264587 10.1016/j.placenta.2012.01.004

[phy270262-bib-0011] Inbal, A. , Skarbianskis, N. , Hochgerner, H. , Ophir, O. , Weiner, Z. , Yagel, S. , Solt, I. , & Zeisel, A. (2023). Two distinct molecular faces of preeclampsia revealed by single cell transcriptomics. Medicine, 4(10), 687–709. 10.1016/j.medj.2023.07.005 37572658

[phy270262-bib-0012] Li, X. , Dai, A. , Tran, R. , & Wang, J. (2023). Identifying miRNA biomarkers for breast cancer and ovarian cancer: A text mining perspective. Breast Cancer Research and Treatment, 201, 5–14. 37329459 10.1007/s10549-023-06996-y

[phy270262-bib-0013] Liem, D. A. , Murali, S. , Sigdel, D. , Shi, Y. , Wang, X. , Shen, J. , Choi, H. , Caufield, J. H. , Wang, W. , Ping, P. , & Han, J. (2018). Phrase mining of textual data to analyze extracellular matrix protein patterns across cardiovascular disease. American Journal of Physiology. Heart and Circulatory Physiology, 315, H910–H924. 29775406 10.1152/ajpheart.00175.2018 PMC6230912

[phy270262-bib-0014] National Institutes of Health . (2024). Mesh browser. U.S. National Library of Medicine. https://meshb.nlm.nih.gov/treeView

[phy270262-bib-0015] Rhein, A. K. , Rabinovich, A. , Abuhasira, R. , Lubaton‐Barshishat, S. , & Erez, O. (2024). Obstetric antiphospholipid syndrome carries an increased lifetime risk for obstetric and thrombotic complications‐a population‐based study. Research and practice. Thrombosis and Haemostasis, 8(4), 102430. 10.1016/j.rpth.2024.102430 PMC11127162 38798792

[phy270262-bib-0016] Rzeszotarska, E. , Sowinska, A. , Stypinska, B. , Lutkowska, A. , Felis‐Giemza, A. , Olesinska, M. , Puszczewicz, M. , Majewski, D. , Jagodzinski, P. P. , Haładyj, E. , & Paradowska‐Gorycka, A. (2022). IL‐1β, IL‐10 and TNF‐α polymorphisms may affect systemic lupus erythematosus risk and phenotype. Clinical and Experimental Rheumatology, 40(9), 1708–1717. 35084314 10.55563/clinexprheumatol/qdgq0v

[phy270262-bib-0017] Schuermans, A. , Truong, B. , Ardissino, M. , Bhukar, R. , Slob, E. A. W. , Nakao, T. , Dron, J. S. , Small, A. M. , Cho, S. M. J. , Yu, Z. , Hornsby, W. , Antoine, T. , Lannery, K. , Postupaka, D. , Gray, K. J. , Yan, Q. , Butterworth, A. S. , Burgess, S. , Wood, M. J. , … Honigberg, M. C. (2024). Genetic associations of circulating cardiovascular proteins with gestational hypertension and preeclampsia. JAMA Cardiology, 9(3), 209–220. 38170504 10.1001/jamacardio.2023.4994 PMC10765315

[phy270262-bib-0018] Shazly, S. A. , Trabuco, E. C. , Ngufor, C. G. , & Famuyide, A. O. (2022). Introduction to machine learning in obstetrics and gynecology. Obstetrics & Gynecology, 139, 669–679. 35272300 10.1097/AOG.0000000000004706

[phy270262-bib-0019] Tao, F. , Zhuang, H. , Yu, C. W. , Wang, Q. , Cassidy, T. , Kaplan, L. M. , Voss, C. R. , & Han, J. (2016). Multi‐dimensional, phrase‐based summarization in text cubes. IEEE data. Engineering Bulletin, 39(3), 74–84.

[phy270262-bib-0020] Thadhani, R. , Lemoine, E. , Rana, S. , Costantine, M. M. , Calsavara, V. F. , Boggess, K. , Wylie, B. J. , Moore Simas, T. A. , Louis, J. M. , Espinoza, J. , Gaw, S. L. , Murtha, A. , Wiegand, S. , Gollin, Y. , Singh, D. , Silver, R. M. , Durie, D. E. , Panda, B. , Norwitz, E. R. , … Kilpatrick, S. (2022). Circulating Angiogenic factor levels in hypertensive disorders of pregnancy. NEJM Evidence, 1(12), EVIDoa2200161. 38319832 10.1056/EVIDoa2200161

[phy270262-bib-0021] Urra, M. , Lyons, S. , Gabriela, T. C. , Burwick, R. , & Java, A. (2024). Thrombotic microangiopathy in pregnancy: current understanding and management strategies kidney international reports. Kidney International Reports, 9(8), 2353–2371. 10.1016/j.ekir.2024.05.016 39156177 PMC11328568

[phy270262-bib-0022] Voskamp, M. , Vinhoven, L. , Stanke, F. , Hafkemeyer, S. , & Nietert, M. M. (2022). Integrating text mining into the curation of disease maps. Biomolecules, 12, 12. 10.3390/biom12091278 PMC9496510 36139119

[phy270262-bib-0023] Vuori, M. A. , Kiiskinen, T. , Pitkanen, N. , Kurki, S. , Laivuori, H. , Laitinen, T. , Mäntylahti, S. , Palotie, A. , FinnGen , & Niiranen, T. J. (2023). Use of electronic health record data mining for heart failure subtyping. BMC Research Notes, 16, 208. 37697398 10.1186/s13104-023-06469-x PMC10496250

[phy270262-bib-0024] Williams, P. J. , & Broughton Pipkin, F. (2011). The genetics of pre‐eclampsia and other hypertensive disorders of pregnancy. Best Practice & Research. Clinical Obstetrics & Gynaecology, 4, 405–417. 10.1016/j.bpobgyn.2011.02.007 PMC3145161 21429808

[phy270262-bib-0025] Wu, P. , Green, M. , & Myers, J. E. (2023). Hypertensive disorders of pregnancy. BMJ, 381, e071653. 37391211 10.1136/bmj-2022-071653

