# Supplementary material for: Text phrase‐mining in identifying and classifying maternal proteins and genes across preeclampsia and similar pathologies
Source: Physiol Rep. 2025 Mar 18;13(6):e70262. doi: 10.14814/phy2.70262 (PMC11919630; doi:10.14814/phy2.70262)
Supplement: Supplementary file 1 — Appendix S1. [file PHY2-13-e70262-s001.docx]

Supplementary Material Document 1: Complete List of Maternal Proteins and Genes (MPG)

1. cytochrome_p450_2c9
2. guanine_nucleotide-
3. binding_protein_g
4. beta-2-glycoprotein_1
5. mineralocorticoid_receptor
6. amyloid_beta_a4_protein
7. tissue-type_plasminogen_activator
8. interleukin-6
9. testosterone
10. collagen_alpha-1
11. alpha-galactosidase_a
12. serine_threonine-protein_kinase_mtor
13. neurogenic_locus_notch_homolog_protein_3
14. prostaglandin_g_h_synthase_2
15. metalloproteinase_inhibitor_1
16. estrogen_receptor
17. angiotensinogen
18. p2y_purinoceptor_12
19. complement_c3
20. endothelin-1
21. natriuretic_peptides_b
22. interleukin-4
23. interleukin-8
24. interleukin-10
25. interleukin-1_receptor_antagonist_protein
26. plasminogen_activator_inhibitor_1
27. von_willebrand_factor
28. resistin
29. platelet_glycoprotein_4
30. angiotensin-converting_enzyme
31. erythropoietin
32. methionine_synthase
33. brain-derived_neurotrophic_factor
34. prothrombin
35. nitric_oxide_synthase_endothelial
36. adiponectin
37. 72_kda_type_iv_collagenase
38. matrix_metalloproteinase-9
39. tissue_factor
40. msahepatic_triacylglycerol_lipase
41. c-c_chemokine_receptor_type_2
42. nicotinamide_phosphoribosyltransferase
43. osteopontin
44. toll-like_receptor_4
45. tumor_necrosis_factor
46. interstitial_collagenase
47. arachidonate_5-lipoxygenase-activating_protein
48. p-selectin
49. mygceru
50. interferon_gamma
51. interleukin-17a
52. myeloperoxidase
53. endothelin-1_receptor
54. heme_oxygenase_1
55. chitinase-3-like_protein_1
56. neurogenic_locus_notch_homolog_protein_1
57. apolipoprotein_a-v
58. cd40_ligand
59. transthyretin
60. adm_cleaved_into_adrenomedullin
61. cytochrome_p450_2c19
62. nitric_oxide_synthase_inducible
63. rac-alpha_serine_threonine-protein_kinase
64. methylenetetrahydrofolate_reductase
65. myeloblastin
66. intercellular_adhesion_molecule_1
67. stromal_cell-derived_factor_1
68. fincprotein
69. jagged-1
70. protein_s100-b
71. thrombomodulin
72. coagulation_factor_viii
73. retinol-binding_protein_4
74. angiopoietin-2
75. vascular_endothelial_growth_factor_receptor_1
76. tyrosine-protein_phosphatase_non-receptor_type_11
77. endothelial_lipase
78. oxidized_low-density_lipoprotein_receptor_1
79. ceramides
80. peroxisome_proliferator-activated_receptor_gamma
81. e-selectin
82. integrin_alpha-iib
83. c-x-c_chemokine_receptor_type_4
84. platelet_glycoprotein_vi
85. haptoglobin
86. c-c_motif_chemokine_2
87. vascular_cell_adhesion_protein_1
88. hepatocyte_growth_factor
89. leptin
90. leptin_receptor
91. tyrosine-protein_kinase_jak2
92. apolipoprotein_e
93. troponin_i_cardiac_muscle
94. renin
95. stm1
96. vascular_endothelial_growth_factor_a
97. 3-hydroxy-3-methylglutaryl-coenzyme_a_reductase
98. nuclear_factor_erythroid_2-related_factor_2
99. glucocorticoid_receptor
100. c-c_chemokine_receptor_type_5
101. alpha-2-hs-glycoprotein
102. vascular_endothelial_growth_factor_receptor_2
103. toll-like_receptor_2
104. interleukin-1_beta
105. angiotensin-converting_enzyme_2
106. lipoprotein_lipase
107. interleukin-18
108. galectin-3
109. gap_junction_alpha-1_protein
110. matrix_gla_protein
111. c-c_motif_chemokine_5
112. signal_transducer_and_activator_of_transcription_3
113. neutrophil_gelatinase-associated_lipocalin
114. cytochrome_b-245_light_chain
115. tissue_factor_pathway_inhibitor
116. estrogen_receptor_beta
117. cx3c_chemokine_receptor_1
118. natriuretic_peptides_a
119. platelet-activating_factor_acetylhydrolase
120. insulin-like_growth_factor_i
121. integrin_beta-3
122. stromelysin-1
123. transcription_factor_7-like_2
124. tumor_necrosis_factor_receptor_superfamily_member_11b
125. mannose-binding_protein_c
126. type-2_angiotensin_ii_receptor
127. endoglin
128. fumh
129. low-density_lipoprotein_receptor
130. macrophage_migration_inhibitory_factor
131. elastin
132. mitochondrial_uncoupling_protein_2
133. peroxisome_proliferator-activated_receptor_alpha
134. pappalysin-1
135. carboxypeptidase_b2
136. pro-neuropeptide_y_cleaved_into_neuropeptide_y
137. coagulation_factor_xiii_a_chain
138. transforming_growth_factor_beta-1
139. scavenger_receptor_class_b_member_1
140. beta-1_adrenergic_receptor
141. coagulation_factor_v
142. tumor_necrosis_factor_ligand_superfamily_member_11
143. lymphotoxin-alpha
144. platelet_glycoprotein_ib_alpha_chain
145. type-1_angiotensin_ii_receptor
146. apolipoprotein_a-i
147. interleukin-1_alpha
148. tumor_necrosis_factor_receptor_superfamily_member_1a
149. leg1
150. fatty_acid-binding_protein_adipocyte
151. sodium_channel_protein_type_9_subunit_alpha
152. fatty_acid-binding_protein_heart
153. n_aa
154. apelin
155. beta-2_adrenergic_receptor
156. integrin_alpha-2
157. endothelial_protein_c_receptor
158. thrombospondin-1
159. proprotein_convertase_subtilisin_kexin_type_9
160. hypoxia-inducible_factor_1-alpha
161. cholesteryl_ester_transfer_protein
162. cystathionine_beta-synthase
163. vitamin_k-dependent_protein_c
164. taglfibrillin-1
165. coagulation_factor_viiia
166. antithrombin-iii
167. troponin_t_cardiac_muscle
168. acylcarnitine
169. complement_factor_h
170. a_disintegrin_and_metalloproteinase_with_thrombospondin_motifs_13
171. hemoglobin_subunit_alpha
172. mitogen-activated_protein_kinase_1
173. cystatin-c
174. appetite-regulating_hormone
175. platelet_endothelial_cell_adhesion_molecule
176. b2_bradykinin_receptor
177. hcdhalpha-adducin
178. metalloproteinase_inhibitor_2
179. advanced_glycosylation_end_product-specific_receptor
180. apolipoprotein_b-100
181. serine_threonine-protein_kinase_receptor_r3
182. fatty_acid-binding_protein_intestinal
183. monocyte_differentiation_antigen_cd14
184. phosphatidylcholine-sterol_acyltransferase
185. vitamin_k_epoxide_reductase_complex_subunit_1
186. tgf-beta_receptor_type-1
187. methionine_synthase_reductase
188. ryanodine_receptor_2
189. moesa2
190. apcytochrome_p450_11b2_mitochondrial
191. plasma_protease_c1_inhibitor
192. prolow-density_lipoprotein_receptor-related_protein_1
193. vitamin_d3_receptor
194. fibroblast_growth_factor_23
195. myosin-7
196. cytotoxic_t-lymphocyte_protein_4
197. cardiac_phospholamban
198. krev_interaction_trapped_protein_1
199. serum_paraoxonase_arylesterase_2
200. serum_paraoxonase_arylesterase_1
201. coagulation_factor_xiii_a_chain
202. grp75
203. atp-binding_cassette_sub-family_a_member_1
204. nucg
205. sodium_channel_protein_type_5_subunit_alpha
206. tgf-beta_receptor_type-2
207. apolipoprotein_c-iii
208. cellular_tumor_antigen_p53
209. interleukin-6_receptor_subunit_alpha
210. glucokinase_regulatory_protein
211. integrin_beta-1
212. high_mobility_group_protein_b1
213. apolipoprotein_c-ii
214. inward_rectifier_potassium_channel_2
215. plakophilin-2
216. cyclin-dependent_kinase_inhibitor_2a_isoforms_1_2_3
217. gamma-glutamyltranspeptidase_1
218. superoxide_dismutase_mn_mitochondrial
219. titin
220. fibrinogen_gamma_chain
221. t-box_transcription_factor_tbx5
222. est1
223. transcription_factor_gata-4
224. glutathione_s-transferase_theta-1
225. tropomyosin_alpha-1_chain
226. serine-protein_kinase_atm
227. peroxisome_proliferator-activated_receptor_gamma_coactivator_1-alpha
228. tumor_necrosis_factor_receptor_superfamily_member_5
229. nduv1
230. myosin-binding_protein_c_cardiac-type
231. potassium_voltage-gated_channel_subfamily_h_member_2
232. potassium_voltage-gated_channel_subfamily_e_member_2
233. potassium_voltage-gated_channel_subfamily_e_member_1
234. potassium_voltage-gated_channel_subfamily_kqt_member_1
235. ndu2
236. hsp74
237. homeobox_protein_nkx-2_5
238. catechol_o-methyltransferase
239. cyclin-dependent_kinase_4_inhibitor_b
240. FLT-1
241. PlGF
242. VEGF
243. vWF
244. ADAMTS-13
245. C5a
246. C5b-9
247. SFLT
248. VEGF
249. PIGF
250. TGF b
251. Relaxin
252. HO-1
253. Angiotensin
254. Complement
